# Application of an Electrochemical Immunosensor with a MWCNT/PDAA Modified Electrode for Detection of Serum Trypsin

**DOI:** 10.3390/s140610203

**Published:** 2014-06-10

**Authors:** Qiang Yi, Qicai Liu, Feng Gao, Qingquan Chen, Guina Wang

**Affiliations:** 1 Department of Laboratory Medicine, the 1st Affiliated Hospital, Fujian Medical University, Fuzhou 350005, China; E-Mail: yiqiang8798@163.com; 2 Department of Pathology, the 1st Affiliated Hospital, Fujian Medical University, Fuzhou 350005, China; E-Mail: fengfang77777@163.com; 3 Department of Laboratory Medicine, College of Medical Technology and Engineering, Fujian Medical University, Fuzhou 350005, China; E-Mails: cqq036@fjmu.edu.cn (Q.C.); wangguina8798@163.com (G.W.)

**Keywords:** electrochemical immunosensor, trypsin, multiwalled carbon nanotubes, nanogold

## Abstract

*Objective*: To establish an electrochemical immunosensor for the determination of serum trypsin levels using a multiwall carbon nanotubes (MWCNTs)-composite-modified electrode. *Method*: A MWCNT composite coated on the surface of bare gold electrodes was used for fixation of an anti-trypsin antibody. The assembly process and the performance indicators, including sensitivity, linear range of detection, anti-jamming performance, and stability, of the electrochemical immunosensor were examined by cyclic voltammetry (CV) and electrochemical impedance spectroscopy (EIS). *Results*: With optimized experimental conditions, the difference of the current value measured by differential pulse voltammetry (DPV) showed a linear relationship with the concentration of serum trypsin within 0.10–100 ng/mL. The detection limit for trypsin using this sensor was 0.002 ng/mL. *Conclusions*: The electrochemical immunosensor built using the MWCNT-composite-modified electrode is simple to operate and has a fast response time, along with a wide linear range, high sensitivity, and accuracy, making it suitable for serum trypsin detection.

## 1. Introduction

Pancreatic cancer is a common cancer with a high mortality rate. Due to a lack of early diagnostic and prognostic markers, more than 80% of clinically-confirmed pancreatic cancers are diagnosed in the later stages, limiting the availability of treatment options [1–3]. Early diagnosis is the key to improve the prognosis of pancreatic cancer, but there is still a lack of clinically effective non-invasive screening methods. Therefore, the identification of new serum markers that can facilitate early diagnosis of pancreatic cancer is particularly important.

Trypsin is a digestive enzyme that selectively hydrolyzes polypeptide chains of lysine or arginine residues, and also participates in the invasion and metastasis of pancreatic cancer by promoting the degradation of the extracellular matrix. In addition, trypsin can also activate protease activated receptor 2 (PAR-2) to stimulate pancreatic cancer cell proliferation and adhesion. Therefore, pancreatic trypsin in serum may be useful as a marker for pancreatic cancer [4–8]. New, highly sensitive, and non-invasive methods for detection of serum trypsin are urgently needed to enable monitoring for high-risk individuals.

At present, the serum trypsin detection technology of choice is ELISA, so development of a rapid, accurate, low cost technology to detect serum trypsin is urgently needed. However, these methods are multiple-step processes and it is possible to get false positive results. An electrochemical immunosensor is a sensor that combines immunological and electrochemical technologies for detecting an antigen. Therefore, it possesses the advantages of an electrochemical sensor, including high sensitivity and low cost, as well as that of immunological analysis, including high selectivity, specificity, and low detection limits [9–13]. Compared with primary methods, electrochemical approaches are attractive for biomarker detection because of their specificity, simplicity, and high-throughput. Electrochemical immunosensors are wildly applied in clinical diagnostic tests. In this study, we constructed a novel electrochemical immunosensor for detecting trypsin and investigated its linear range, specificity, stability, and other performance indicators, with the ultimate aim of developing a sensor for the clinical detection of trypsin.

## 2. Methods

### 2.1. Preparation of Poly(diallyldimethylammonium Chloride) (PDDA)-Multiwalled Carbon Nanotubes (MWCNTs)

#### 2.1.1. Preparation of MWCNT-Composite

Two grams of MWCNTs (with a purity of 95%, inside diameter of 10 nm, 5–15 μm length, ash content, 0.2 wt%, and a unit surface area of 40–300 m^2^/g; Nano-Tech Port, Shenzhen, People's Republic of China) were repeatedly rinsed in concentrated hydrochloric acid (250 mL) for 10 h, and then cooled to room temperature and washed with distilled water until the dispersion reached neutral pH. Then, a mixture of concentrated nitric acid and concentrated sulfuric acid (400 mL, 1:3, v/v) was added to the MWCNTs; the dispersion was sonicated for 8 h and washed with distilled water until it reached neutral pH. The nanotubes were separated by centrifugation and then dried in an oven at 120 °C. The MWCNTs solution (1 g/L) was prepared by adding the treated MWCNTs (10 mg) to borate buffer solution (10 mL, pH 9.1), followed by sonication for 30 min.

#### 2.1.2. Preparation of PDDA-MWCNTs Dispersion

PDDA-MWCNTs solution (1 g/L) was prepared by adding MWCNTs (1 mg) to PDDA solution (1 mL, 10 g/L; Sigma, SAINT LOUIS, MO, USA), followed by sonication for 60 min to form a homogenous dark solution, which was kept at room temperature for 24 h, filtered, and then stored at 4 °C until further use.

#### 2.1.3. Characterization of the Electrochemical Behavior of the Modified Electrode in Acidic Aqueous Solution by Cyclic Voltammetry

We used the modified AuE as the working electrode, a saturated calomel electrode as the reference electrode, and a platinum wire electrode as the auxiliary electrode. The working electrode was immersed in phosphate-buffered saline (PBS, pH 7.4) and its detection efficiency was tested using stripping voltammetry (potential range of scan, +0.4 to −0.5 V; scanning speed, 100 mV/s). The sensitivity and liner range of trypsin detection, as well as anti-jamming performance and stability, of the electrochemical immunosensor were evaluated.

#### 2.1.4. Comparison of Enzyme-Linked Immunosorbant Assay (ELISA)-Based and Electrochemical Immunosensor-Based Detection of Trypsin

The tryspin concentration in serum samples, with concentrations of 80.63, 42.31, and 8.53 ng/mL trypsin as determined using an ELISA kit (Boyanbio, Shanghai, China), were re-analyzed using the electrochemical immunosensor. The results obtained from the two different analytic methods were compared to determine the accuracy and sensitivity of the electrochemical immunosensor.

### 2.2. Preparation of the Electrochemical Immunosensor

#### 2.2.1. Pretreatment of Gold Electrodes

Gold electrodes (Shanghai Chenhua Instruments, Shanghai, People's Republic of China) were polished on suede with 0.3 μm Al_2_O_3_ powder and then subsequently sonicated in nitric acid (concentrated nitric acid:H_2_O = 1:1), anhydrous ethanol, and ultra-pure water for 2 min, followed by drying under a stream of gaseous nitrogen. The electrodes were continuously scanned with a potential of −0.4–1.6 V to steady state (scanning speed, 100 mV/s) in 0.1 mol/L sulfuric acid solutions. They were then washed with ultra-pure water and dried under a stream of gaseous nitrogen.

#### 2.2.2. Preparation of Antibody-Modified Gold Electrodes

**Figure 1.**
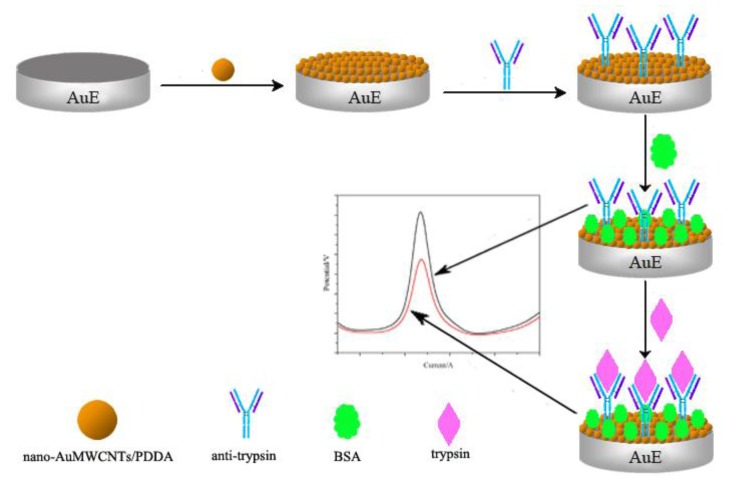
The preparation and assembly procedure for generating the immunosensor.

The processed electrode was immersed in 1 g/L of PDDA-MWCNTs solution for 30 min and then washed with distilled water and dried under a stream of gaseous nitrogen. Then, the PPDA-MWCNT-coated electrodes were immersed in gold nanoparticle solution for 30 min, followed by washing with ultra-pure water to remove unbound gold nanoparticles and then dried under a steam of gaseous nitrogen to generate the nano-gold (Au)-MWCNTs/PDDA composite-modified electrode (AuE). The AuE was then coated with 6 μL of an anti-trypsin antibody (Ab1; Sangong Biotech, Shanghai, People's Republic of China) at 4 °C for 16 h, followed by blocking with 2% bovine serum albumin (Sangong Biotech) solution at room temperature for 2 h. The AuEs were then washed with PBS and dried under a stream of gaseous nitrogen to generate the Ab1-nano-Au-MWCNTs/PDDA (Ab1-AuE). The preparation and assembly procedure for generating the immunosensor is shown in Figure 1.

## 3. Results

### 3.1. Electrochemical Characteristics of the Electrochemical Immunosensor

**Figure 2.**
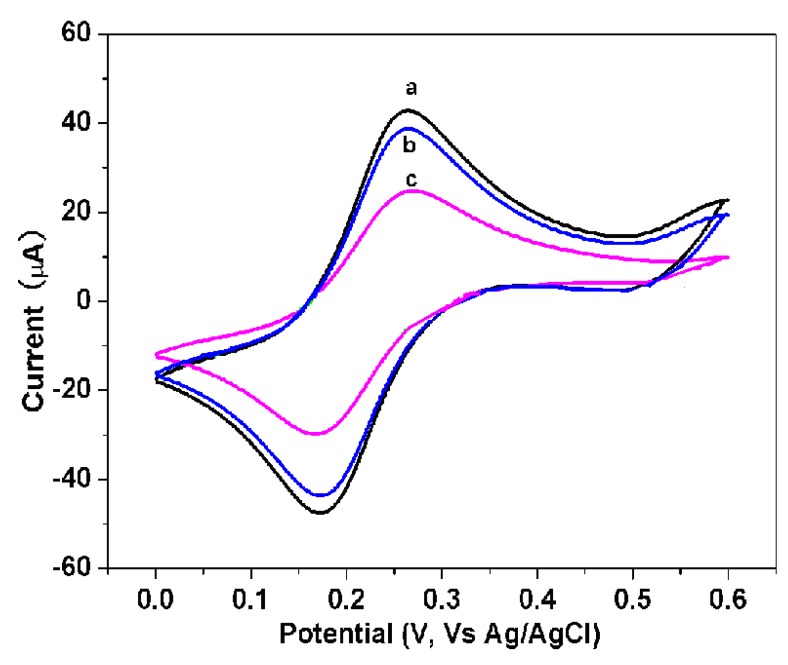
Different steps of cyclic voltammograms of nano-gold (Au)-MWCNTs/PDDA composite-modified electrode.

We used the modified AuE as the working electrode, a saturated calomel electrode as the reference electrode, and a platinum wire electrode as the auxiliary electrode. The cyclic voltametry curve was recorded in the voltage range of 0.0–0.6 V in 0.1 mol/L potassium chloride solution containing 5.0 mmol/L [Fe(CN)_6_]^3−/4−^ (scanning speed 100 mV/s) (Figure 2). The nano-Au-MWCNTs/PDDA composite membrane-covered electrode possessed a pair of reversible redox/oxidation peaks (curve a, Figure 2). When the anti-trypsin antibody adsorbed to the surface of the composite membrane, the current decreased (curve b, Figure 2). When the trypsin bound to the anti-trypsin antibody fixed on the electrode, the current decreased further (curve c, Figure 2).

**Figure 3.**
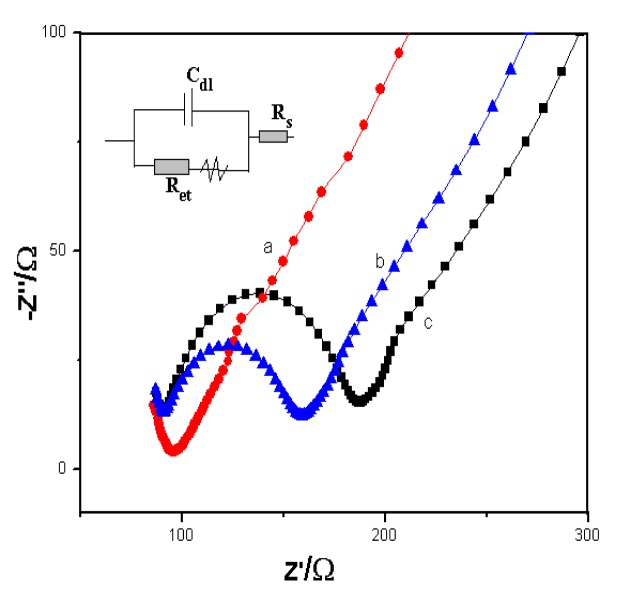
Electrochemical impedance spectroscopy (EIS), the change of electron transfer resistance (Ret) value on the AuE (a), Ab1-AuE (b), and antigen-bound Ab1-AuE (c) electrodes in 0.1 mol/L potassium chloride solution with 5.0 mmol/L [Fe(CN)_6_]^3−/4−^.

Electrochemical impedance spectroscopy (EIS): we monitored the change of electron transfer resistance (Ret) value on the AuE, Ab1-AuE, and antigen-bound Ab1-AuE electrodes in 0.1 mol/L potassium chloride solution with 5.0 mmol/L [Fe(CN)_6_]^3−/4−^. The electron transmission of [Fe(CN)_6_]^3−/4−^ on the modified electrode possessed a small diameter, indicating that the electrons on the surface of AuE could be quickly transferred (curve a, Figure 3). When the anti-trypsin antibody absorbed to the surface of electrode, the resistance value increased dramatically (curve b, Figure 3); this was attributable to the absorbed antibody, which would impede the electron transmission, onto electrode surface. These data also indicated that the antibody was successfully absorbed onto the surface of the electrode. After trypsin bound to the antibody-modified electrode, the resistance value was further increased (curve c, Figure 3). The AC impedance and cyclic voltammetry curves of different modified electrodes were consistent, indicating the successful preparation of the electrochemical immunosensor.

### 3.2. Performance Study of the Electrochemical Immunosensor

#### 3.2.1. Sensitivity and Accuracy

**Figure 4.**
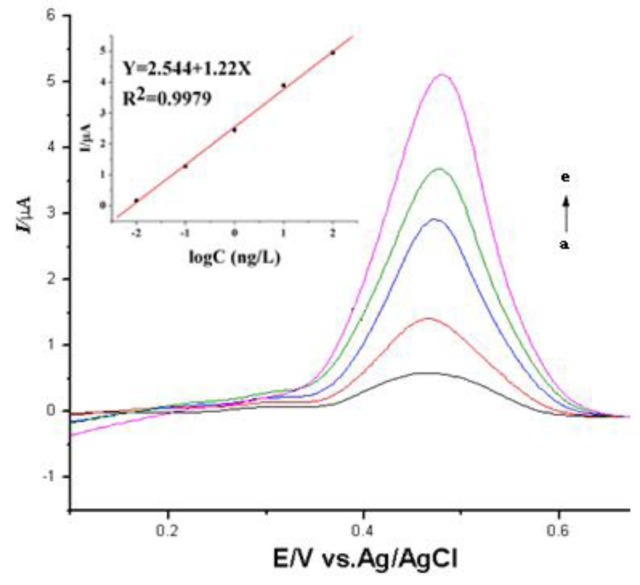
The decrease in anodic currents subtracted from the sameconditions of PBS substance was proportional to the logarithm of the trypsin concentrations, a→e indicate of the trypsin concentrations 0.01 ng, 0.1 ng, 1 ng, 10 ng, and 100 ng/mL.

The sensitivity and the linearity of trypsin detection by the electrochemical immunosensor were evaluated by voltammetry using different concentrations of a trypsin standard (0.01, 0.1, 1, 10, and 100 ng/mL). The differential current between the antibody-modified and antigen-bound antibody-modified electrodes increased as the concentrations of trypsin increased (Figure 4). The plot of the current value (I) and the logarithm of the concentration of trypsin (logC) fit well to a linear regression within the trypsin concentration range of 0.1–100 ng/mL. The equation for the linear regression was: I (μA) = 2.544 + 1.22 logC (ng/L), with an r^2^ = 0.9979 and a detection limit = 0.002 ng/mL (signal/noise [S/N] = 2). Parallel measurement of the differential currents in the presence of 0.05 ng/mL trypsin was repeated five times (the relative standard deviation was 5.6%) to confirm the consistent performance of the electrochemical immunosensor.

#### 3.2.2. Anti-Jamming Experiment

**Figure 5.**
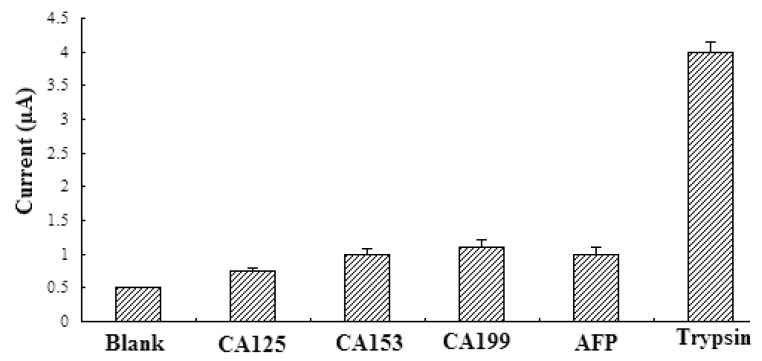
The specificity of the immunosensor tested the CV responsesto some common interfering proteins. Its CV responses to CA125, CA153, CA199, and trypsin were investigated under the same experimental conditions.

The ability of the sensor to detect trypsin in complex mixtures was tested first by using a mixture of trypsin and other tumor marker. Trypsin samples mixed with several common tumor markers (including carbohydrate antigens CA125, CA199, CA153, and α-fetoprotein [AFP]) at concentrations 10–1000 times higher than that of trypsin were tested using a differential pulse voltamtery (DPV) assay. The signals of other tumor markers were markedly smaller than that of trypsin (Figure 5). The interference signals of the CA125, CA153, CA199, and AFP proteins were close to that of a blank solution, lacking any proteins, which indicated the high selectivity of the electrochemical immunosensor.

#### 3.2.3. Stability of Electrochemical Immunosensor

After fixation of the anti-trypsin antibody, the electrode of the electrochemical immunosensor constructed in this study could be stored in PBS at 4 °C for 2 weeks. The signal was relatively stable and could be maintained at 90% consistency between different electrochemical immunosensors manufactured by the same method, indicating long-term stability.

#### 3.2.4. Determination of Serum Samples

The trypsin samples (containing 80.63, 42.31, and 8.53 ng/mL trypsin, as determined by ELISA) were examined using the electrochemical immunosensor. Results at 4.55, 3.96, and 3.29 μA, showed the differential current value increased as the concentration of the trypsin increased. According to the principle that the trypsin amount is inversely proportional to the current value, we can clearly distinguish the high, median, and low concentrations of the trypsin. These results demonstrated that the electrochemical immunosensor established in this study possessed sample measurement capacity suitable for practical applications.

## 4. Discussion

Electrochemical immunosensors are constructed based on the combination of the principle of the antibody/antigen reaction and the electrochemical sensing method. Such sensors possessed many advantages, including high sensitivity, fast response times, simplicity in operative procedures, and suitability for miniaturization. Currently, electrochemical immunosensors have been widely used to detect tumor markers, pesticide residues, and bacterial toxins [12–15]. The critical factors in electrochemical immunoassay are the amount and the affinity of fixed biomolecules on the immunosensor, and whether the antibody or antigen in the sample is capable of binding readily with the biomolecules on the electrode. Biomolecules immobilized on the electrode surface by the embedding method can enhance the stability of the biomolecules on the sensor, preventing its leakage. But, the disadvantage of this method for generating sensors is the increase in the allosteric hindrance between the antigen or antibody in the sample and the biomolecules on the electrode. Some researchers have attempted to circumvent this issue by fixing the biomolecules using chemical bonding, which not only increases the stability of the fixation, but also reduces steric hindrance. However, this approach might lead to a molecular conformation change and reduced biological activity of the fixed biomolecules [16–19]. Based on the advantages and disadvantages of these two fixation methods, in this study, we used combined the biological analog interface and self-assembly technology to fix the biomolecules onto the sensor surface, which preserved the spatial conformation and the biological activity of the biomolecules.

In this study, we immobilized the anti-trypsin antibody on the surface of the MWCNT-gold nanoparticle-modified electrode. The MWCNTs and the nano-potassium ferricyanide have an expanded electrode surface and enhanced bio-compatibility, which make the modified electrode surface absorb larger amounts of antibodies and maintain significant biological activity. Therefore, the sensitivity and stability of the electrodes was greatly improved. When detecting various concentrations of trypsin, the electrochemical immunosensor showed an increase of the differential current value concomitant with the increase in trypsin concentrations. Within the trypsin concentration range of 0.1–100 ng/mL, the differential current value (I) showed a good linear relationship with the logarithm of the trypsin concentrations (logC). The detection limit was 0.002 ng/mL and the measurement was consistent at low concentrations of trypsin. After fixing the anti-trypsin antibody, the immunosensor could be stored in PBS at 4 °C for 2 weeks, while still retaining 90% of the original signal, indicating its good stability.

## 5. Conclusions

We have demonstrated a sensor possessing high sensitivity, stability, and reproducibility. The results of measurements using clinical samples also demonstrated the potential of this electrochemical immunosensor in practical applications and provide a basis for further development of this technology in clinical applications.
